# Lower serum 15-HETE level predicts nasal ILC2 accumulation during COX-1 inhibition in AERD

**DOI:** 10.1016/j.jaci.2023.06.028

**Published:** 2023-08-03

**Authors:** Jana H. Badrani, Kellen Cavagnero, Jacqueline J. Eastman, Alex S. Kim, Allyssa Strohm, Carol Yan, Adam Deconde, Bruce L. Zuraw, Andrew A. White, Sandra C. Christiansen, Taylor A. Doherty

**Affiliations:** a Section of Allergy and Immunology, Department of Medicine, University of California San Diego, La Jolla; b Veterans Affairs San Diego Healthcare System, La Jolla; c Department of Otolaryngology, University of California, San Diego; d Divison of Allergy, Asthma and Immunology, Scripps Clinic, La Jolla

**Keywords:** ILC2, innate lymphoid cells, AERD, asthma, nasal polyps, eicosanoid, lipidomic, 15-HETE, 19,20-diHDPA

## Abstract

**Background:**

Aspirin-exacerbated respiratory disease (AERD) is associated with high levels of cysteinyl leukotrienes, prostaglandin D_2_, and low levels of prostaglandin E_2_. Further, 15-hydroxyeicosatetraenoic acid (15-HETE) levels may have predictive value in therapeutic outcomes of aspirin desensitization. Accumulation of nasal group 2 innate lymphoid cells (ILC2s) has been demonstrated during COX-1 inhibition in AERD, although the relationships between tissue ILC2 accumulation, reaction symptom severity, and novel lipid biomarkers are unknown.

**Objective:**

We sought to determine whether novel lipid mediators are predictive of nasal ILC2 accumulation and symptom scores during COX-1 inhibitor challenge in patients with AERD.

**Methods:**

Blood and nasal scraping samples from patients with AERD were collected at baseline and COX-1 inhibitor reaction and then processed for flow cytometry for nasal ILC2s and serum for lipidomic analysis.

**Results:**

Eight patients with AERD who were undergoing aspirin desensitization were recruited. Of the 161 eicosanoids tested, 42 serum mediators were detected. Baseline levels of 15-HETE were negatively correlated with the change in numbers of airway ILC2s (*r* = −0.6667; *P* = .0428). Docosahexaenoic acid epoxygenase metabolite 19,20-dihydroxy-4Z,7Z,10Z,13Z,16Z-docosapentaenoic acid (19,20-diHDPA) was positively correlated with both changes in airway ILC2s (*r* = 0.7143; *P* = .0305) and clinical symptom scores (*r* = 0.5000; *P* = .0081).

**Conclusion:**

Low levels of baseline 15-HETE predicted a greater accumulation of airway ILC2s in patients with AERD who were receiving COX-1 inhibition. Further, increases in the cytochrome P pathway metabolite 19,20-dihydroxy-4Z,7Z,10Z,13Z,16Z-docosapentaenoic acid (19,20-diHDPA) were associated with increased symptoms and nasal ILC2 accumulation. Future studies to assess how these mediators might control ILC2s may improve the understanding of AERD pathogenesis.

## INTRODUCTION

Adult-onset asthma, chronic rhinosinusitis with nasal polyposis, and respiratory reactions to aspirin and other COX-1 inhibitors are characteristic of aspirin-exacerbated respiratory disease (AERD).^1^ AERD is associated with dysregulation of the arachidonic acid metabolic pathways, generating high levels of proinflammatory eicosanoids (prostaglandin D2 [PGD_2_] and cysteinyl leukotrienes [CysLTs]) and low levels of anti-inflammatory eicosanoids (prostaglandin E_2_ and lipoxin A_4_).^2–6^ AERD pathogenesis involves interactions with several immune cellular players, including mast cells, eosinophils, basophils, T cells, and group 2 innate lymphoid cells (ILC2s).^7,8^ CysLTs contribute to the release of epithelial IL-33, which can activate mast cells to induce release of PGD_2_.^9^ PGD_2_ can, in turn, activate eosinophils, basophils, T_H_2 cells, and ILC2s by binding to chemoattractant receptor–homologous molecules expressed on T_H_2 cells (CRTH2). In addition, IL-33 and CysLTs act synergistically to directly activate ILC2s.^10^ Activated ILC2s produce IL-5 and IL-13, which promote eosinophil accumulation, airway hyperresponsiveness, and mucus production.^8^ Thus, eicosanoid lipid mediators and alarmin cytokines that regulate ILC2 recruitment and activation may be central to the pathogenesis of AERD.

Previous lipidomic studies of plasma, urine, and sputum samples from patients with AERD collected throughout the aspirin desensitization have highlighted differences in specific metabolites that may serve as predictive markers of treatment outcomes. For example, serum baseline levels of leukotriene E_4_ (LTE_4_) have been detected at higher levels in patients with AERD.^11^ Recently, Jerschow et al analyzed the plasma of patients with AERD who were undergoing aspirin desensitization and observed that high baseline levels of 15-hydroxyeicosatetraenoic acid (15-HETE) were correlated with more favorable outcomes.^12^ 15-HETE is a product of 15-lipoxygenase (15-LO) and is converted to the anti-inflammatory eicosanoid lipoxin A4.^13^ Importantly, expression of 15-LO has also been demonstrated to be significantly increased in nasal polyps of patients with AERD relative to that in patients with chronic rhinosinusitis.^14^ Thus, there may be a role for 15-HETE and other lipid mediators as biomarkers that may also contribute to AERD pathogenesis.

In the past 15 years, increased levels of activated ILC2s have been detected in samples from patients with type 2 inflammatory diseases, and show to be critical drivers of type 2 inflammation in animal models and in human *in vitro* studies. Previously, we demonstrated that patients with AERD undergoing COX-1 inhibitor challenge and desensitization had increased accumulation of ILC2s in the nasal mucosa during reactions that were additionally correlated with maximum symptom scores and increases in urinary levels of leukotriene E_4_ and PGD_2_ metabolite 11-β-PGF2a.^15^ However, broad lipid analysis linking novel lipid mediators to ILC2 levels in AERD has not been reported. In this study, we performed extensive eicosanoid analysis of peripheral blood from patients with AERD before and during aspirin desensitization and compared mediator levels with nasal ILC2 levels and symptom scores.

## RESULTS AND DISCUSSION

The samples from patients included in this study were from our prior reported study.^15^ A total of 8 patients with AERD who were not previously treated with biologic therapy underwent COX-1 inhibitor challenge and desensitization at the University of California San Diego and Scripps Clinic Allergy Divisions. Patients were confirmed to have AERD based on a history of asthma, nasal polyps, and reactions to aspirin or other nonsteroidal anti-inflammatory drugs, as well as characteristic reactions to COX-1 inhibitor challenge. Nasal scrapings and blood were collected at baseline and at the time of a clinician-confirmed reaction and postreaction time points. ILC2s were identified within nasal scrapings as lineage-negative CRTH2-positive lymphocytes, as we have previously reported.^15,16^ We assessed serum levels of 161 eicosanoid mediators and detected 42 mediators. Lipids associated with distinct pathways of arachidonic acid metabolism, including the lipoxygenase (LOX) and cytochrome P450 (CYP450) pathways and nonenzymatic oxidation, were analyzed and correlated with nasal ILC2 levels.

**FIG 1.**
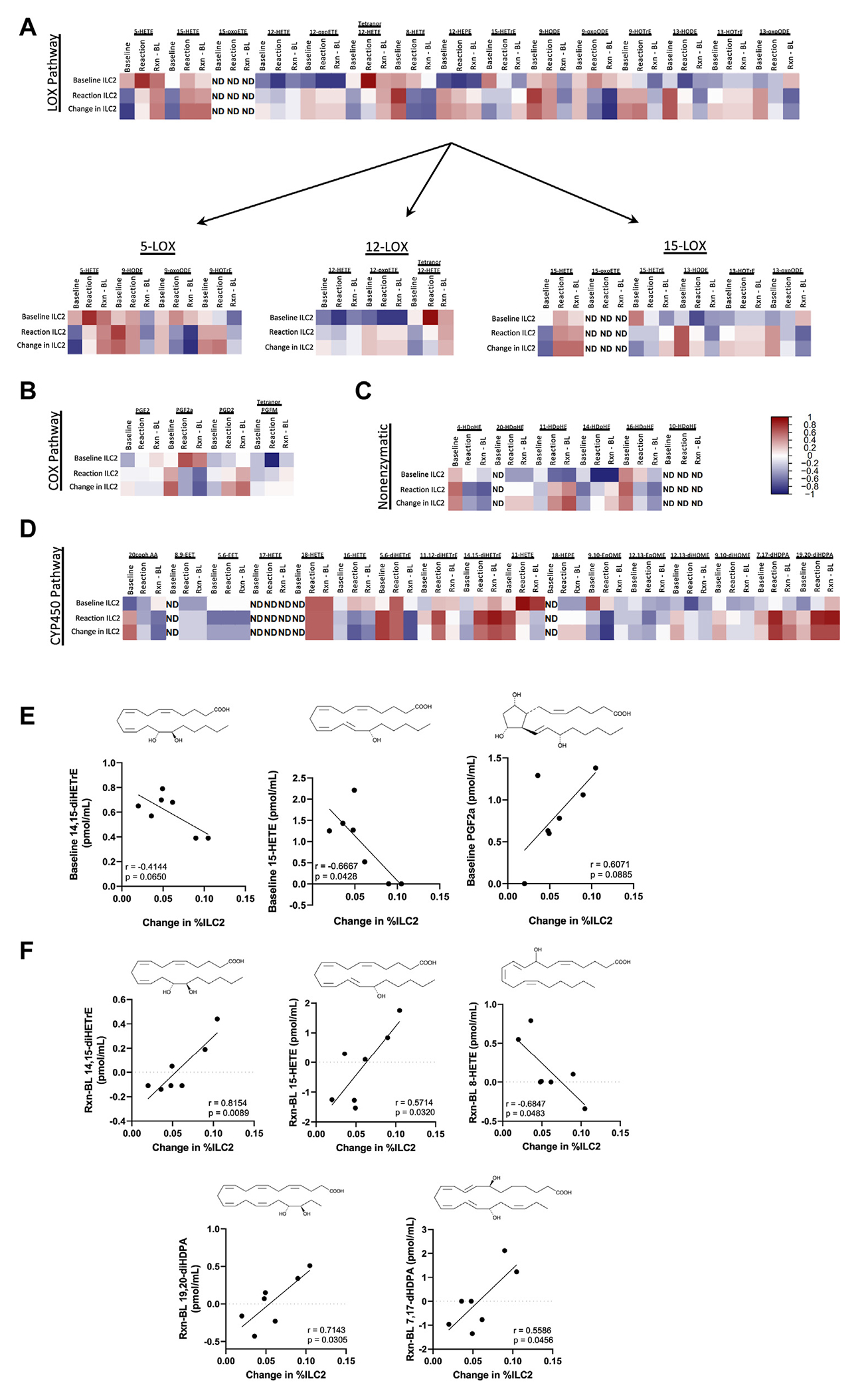
Eicosanoids at baseline (BL) and reaction (Rxn) were correlated with airway ILC2 accumulation following aspirin desensitization. Spearman correlations between lipid mediators and airway ILC2 percentages were calculated before and during COX-1 inhibition. **A-D**, Heatmaps of these correlations separated on the basis of metabolic pathway. **E** and **F,** Linear regressions of correlations of select mediators and ILC2s. *20cooh AA*, 20-Carboxy arachidonic acid; *diHETrE*, dihydroxy-5Z,8Z,11Z-eicosatrienoic acid; *ND*, not detected; *oxoETE*, oxo-eicosatetraenoic acid; *14,15-diHETrE*, 14,15-dihydroxy-5Z,8Z,11Z-eicosatrienoic acid; *15-HETE*, 15S-hydroxy-5Z,8Z,11Z,13E-eicosatetraenoic acid; *PGF2a*, 9S,11R,15S-trihydroxy-5Z,13E-prostadienoic acid; *8-HETE*, 8-hydroxyeicosatetraenoic acid; *19,20-diHDPA*, 19,20-Dihydroxy-4Z,7Z,10Z,13Z,16Z-docosapentaenoic acid; *7,17-dHDPA*, 7,17-dihydroxy-8E,10Z,13Z,15E,19Z-docosapentaenoic acid.

Spearman correlations between the levels of nasal scraping ILC2s and serum lipid mediators at baseline and during COX-1 inhibitor reactions were calculated, and heatmaps were created of LOX, COX, CYP450, and nonenzymatic pathways (Fig 1, A–D). Levels of lipid mediators at baseline, reaction, and changes from baseline to reaction were compared with changes in nasal ILC2 accumulation from baseline to reaction. The correlations between changes in ILC2 nasal scrapping percentages and levels of lipid mediators of the 15-LOX pathway and CYP450 pathway were most significant (Fig 1, A and D). Baseline levels of 15-HETE were negatively correlated with the change in accumulation of nasal ILC2s (*r* = −0.6667; *P* = .0428) (Fig 1, E), and 14,15-dihydroxy-5Z,8Z,11Z-eicosatrienoic acid (14,15-diHETrE) trended similarly (*r* = −0.4144, *P* = .0650). Levels of the PGD_2_ metabolite PGF2a trended toward a positive correlation with ILC2 accumulation, although this trend did not meet statistical significance owing to 1 outlier (*r* = 0.6071; *P* = .0885) (Fig 1, E). Increases in 14,15-dihydroxy-5Z,8Z,11Z-eicosatrienoic acid (14,15-diHETrE) and 15-HETE levels from baseline to reaction positively corresponded with increases in ILC2 accumulation (*r* = 0.8154; *P* = .0089 and *r* = 0.5714; *P* = .0320, respectively) (Fig 1, F). Changes in 8-hydroxyeicosatetraenoic acid (8-HETE) levels were negatively associated with accumulation of airway ILC2s (*r* = −0.6847; *P* = .0483), and the CYP450 pathway mediators 19,20-dihydroxy-4Z,7Z,10Z,13Z,16Z-docosapentaenoic acid (19,20-diHDPA) and 7,17-dihydroxy-8E,10Z,13Z,15E,19Z-docosapentaenoic acid (7,17-dHDPA) positively correlated with nasal ILC2 accumulation (*r* = 0.7143; *P* = .0305 and *r* = 0.5586; *P* = .0456, respectively) (Fig 1, F).

**FIG 2.**
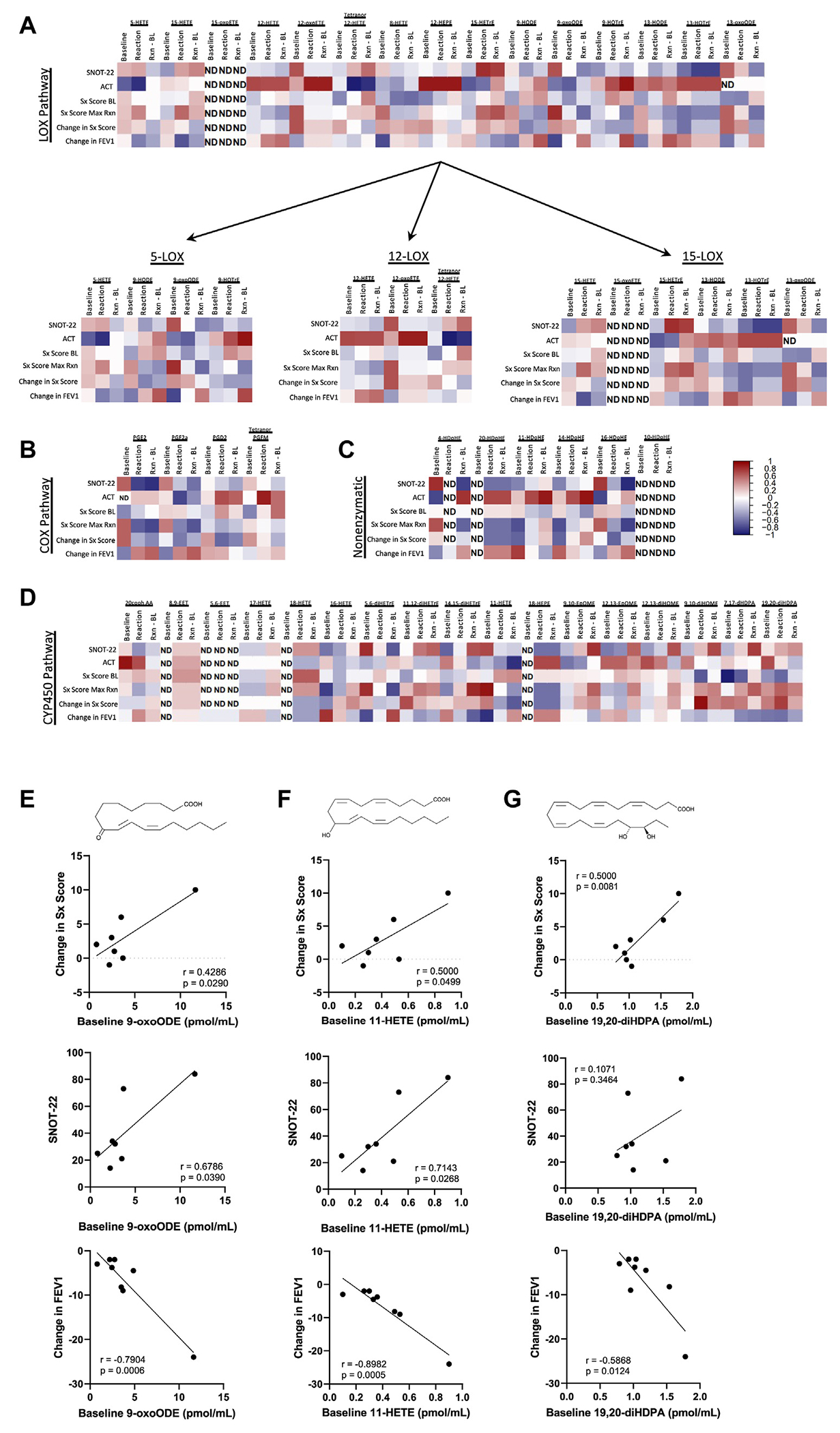
Eicosanoid levels at baseline (BL) and reaction (Rxn) were correlated with symptoms (Sx) during aspirin desensitization. Spearman correlations between lipid mediators and clinical scores were calculated at BL and Rxn. **A-D,** Heatmaps of these correlations separated based on metabolic pathway. **E-G,** Linear regressions of significant correlations. *ACT*, Asthma Control Test; *Max*, maximum; *ND*, not detected; *oxoETE*, oxo-eicosatetraenoic acid; *SNOT-22*, 22-Item Sino-nasal Outcome Test; *9-oxoODE*, 9-oxo-10E,12Z-octadecadienoic acid; *11-HETE*, 11-hydroxyeicosatetraenoic acid; *19,20-diHDPA*, 19,20-Dihydroxy-4Z,7Z,10Z,13Z,16Z-docosapentaenoic acid.

We next explored correlations between lipid mediator levels and clinical symptom assessments in patients with AERD undergoing COX-1 inhibitor challenges. Baseline 22-Item Sino-nasal Outcome Test (SNOT-22) and baseline and reaction AERD symptom scores^15^ and FEV_1_ values were compared with the changes in different arachidonic acid metabolism pathway mediators (Fig 2, A–D). Comparisons between symptom scores and mediators of the LOX pathway and CYP450 pathway demonstrated the strongest correlations. Baseline levels of 9-oxo-10E,12Z-octadecadienoic acid (9-oxoODE), 19,20-diHDPA, and 11-hydroxyeicosatetraenoic acid (11-HETE) were positively associated with a change in symptom score from baseline to reaction (*r* = 0.4286 and *P* = .0290; *r* = 0.500 and *P* = .0081; and *r* = 0.5000 and *P* = .0499, respectively) (Fig 2, E–G). Baseline levels of 9-oxoODE, 19,20-diHDPA, and 11-HETE were negatively correlated with the change in FEV_1_ percentage from baseline to reaction (*r* = −0.7904 and *P* = .0006; *r* = −0.5868 and *P* = .0124; and *r* = −0.8982 and *P* = .0005, respectively). Overall, only 19,20-diHDPA from the CYP450 pathway, was positively correlated with both nasal ILC2 accumulation and symptoms scores as well as with a reduction in FEV_1_ value at reaction. Despite a lack of significant correlations between ILC2 accumulation and baseline 9-oxoODe and 11-HETE levels, these mediators were predictive of clinical outcomes measured following aspirin desensitization and may be of interest for future studies.

Utilizing broad lipidomic analysis, we assessed serum eicosanoid mediator levels at baseline and during COX-1 inhibition, and we correlated the mediator levels with airway ILC2 accumulation and symptom scores. We found that baseline 15-HETE levels were negatively correlated with increases in airway ILC2 accumulation such that lower levels of baseline 15-HETE predicted increased nasal ILC2 accumulation at reaction. Notably, a previous study demonstrated that patients with low levels of 15-HETE at baseline had poorer clinical outcomes after aspirin therapy.^12^ In our study, baseline 15-HETE levels were not associated with symptom scores at reaction, whereas in the previous study 15-HETE levels were correlated with symptoms after 4 weeks of aspirin therapy. It is possible that the rapid accumulation of ILC2s (and possibly other inflammatory cells) in patients with lower 15-HETE might predispose them to worse outcomes at later time points, although this remains to be investigated.

A potential mechanism postulated by Jerschow et al to explain how low levels of 15-HETE might influence clinical outcomes involves the metabolism of 15-HETE to anti-inflammatory lipoxins via the 5-LO pathway.^12,13^ After aspirin treatment, 5-LO converts 15-HETE to the “aspirin-triggered” 15-epimer counterparts of the lipoxins A_4_ and B_4_, which have anti-inflammatory properties in addition to those of the lipoxins A_4_ and B_4_.^17,18^ Intriguingly, lipoxin A_4_ has been demonstrated to directly inhibit human ILC2 activation in the presence of PGD_2_ and IL-33.^19^ Despite this, we did not detect serum lipoxins or resolvins at any time point regardless of symptom severity, although the serum levels may not reflect tissue levels. Thus, it is also possible that 15-HETE might directly or indirectly inhibit chemotaxis of ILC2s, as has been described with inhibition of leukotriene B4–induced neutrophil chemotaxis by 15-HETE.^20^ One limitation of our work is that we confined the eicosanoid analysis to peripheral blood, whereas mediator changes in airway fluid and urine could provide further important insights into lipid changes in patients with AERD.

19,20-diHDPA was the only mediator that was positively correlated with changes in accumulation of nasal scrapping ILC2s, symptom scores, and reduction in FEV_1_ value at reaction. Studies to determine cellular sources of 19,20 di-HDPA are lacking, but a recent report demonstrated production in human monocyte-derived macrophages *in vitro*.^21^ 19,20-diHDPA is metabolized from docosahexaenoic acid, an omega-3 polyunsaturated fatty acid, through the cytochrome P pathway and terminally by diol conversion via soluble epoxide hydrolase (sEH). sEH metabolites such as 19,20-diHDPA are less biologically active than their anti-inflammatory precursors, and inhibition of sEH has been proposed as a therapeutic strategy to reduce airway inflammation.^22–24^ One study demonstrated that low anti-inflammatory lipoxin levels found in patients with severe asthma was due to increased sEH activity and inhibition of sEH increased lipoxin levels.^24^ Further, preclinical studies using sEH inhibitors have demonstrated reductions in airway inflammatory endpoints.^22,23^ The relationship between a potential role of sEH during COX-1 inhibition is largely unexplored, although 1 reported that concomitant inhibition of sEH during aspirin administration in LPS-treated mice reduced serum PGD_2_ level (beyond the level with aspirin alone) and septic outcomes.^25^ Thus, sEH could be investigated further as a potential therapeutic target in AERD. Finally, future studies of the direct or indirect roles of 15-HETE and 19,20 di-HDPA in ILC2 function may provide insights into ILC2 biology and AERD mechanisms.

In summary, we found that baseline 15-HETE levels negatively predict nasal ILC2 accumulation and that levels of 19,20-diHDPA are positively correlated with increases in symptoms and nasal ILC2 accumulation in patients with AERD who are undergoing COX-1 inhibitor challenge. This provides a strong rationale for further studies to understand how novel arachidonic acid pathway mediators regulate ILC2s in AERD pathophysiology.

## Supplementary Material

Suppmethods

## Key messages

- In patients with AERD who are undergoing COX-1 inhibitor challenge, baseline plasma 15-HETE level is negatively correlated with nasal ILC2 accumulation.
- Changes in airway ILC2 levels and clinical scores are positively associated with increases in the docosahexaenoic acid metabolite 19,20-diHDPA.

## Abbreviations used

AERD: Aspirin-exacerbated respiratory disease
CYP450: Cytochrome P450
CysLT: Cysteinyl leukotriene
19,20-diHDPA: 19,20-Dihydroxy-4Z,7Z,10Z,13Z,16Z-docosapentaenoic acid
11-HETE: 11-Hydroxyeicosatetraenoic acid
15-HETE: 15-Hydroxyeicosatetraenoic acid
ILC2: Group 2 innate lymphoid cell
15-LO: 15-Lipoxygenase
LOX: Lipoxygenase
9-oxoODE: 9-Oxo-10E,12Z-octadecadienoic acid
PGD_2_: Prostaglandin D_2_
sEH: Soluble epoxide hydrolase
