# Supplementary material for: Lower serum 15-HETE level predicts nasal ILC2 accumulation during COX-1 inhibition in AERD
Source: J Allergy Clin Immunol. Author manuscript; Available in PMC 2024 Mar 14. (PMC10938261; doi:10.1016/j.jaci.2023.06.028)
Supplement: Suppmethods [file NIHMS1970395-supplement-Suppmethods.pdf]

METHODS

Subjects

This study was approved by the institutional review boards of the University of California San Diego and the Scripps Clinic. The recruited patients had a suspected diagnosis of AERD with a history of asthma, nasal polyps, and reactions to aspirin or other nonsteroidal anti-inflammatory drugs and were referred for aspirin challenge and desensitization. Informed consent was obtained from all patients. Demographic data, clinical history, and medication history were obtained by chart review. The AERD symptom score was generated by using a validated scoring system as detailed in prior studies. In these studies, maximum desensitization symptom scores were positively correlated with baseline 22-Item Sino-Nasal Outcome Test (SNOT-22) scores.[E1]

Challenge and desensitization protocol

Patients were desensitized with intranasal ketorolac and aspirin challenges following the protocol of each institution as described in prior studies.[E1] Following threshold dose reactions, patients were treated until symptom resolution, followed by repeat administration of the threshold dose and continued dose escalation per protocol. Following the Scripps protocol, on the first day of desensitization, patients were challenged with increasing doses of ketorolac from 1.26 mg to 7.58 mg, followed by challenge with 2 doses of either 60 mg or 81 mg of aspirin given 90 minutes apart. On the second day, patients were given 150 mg of aspirin, followed by 325 mg of aspirin. The University of California San Diego protocol included challenges with increasing doses of ketorolac from 1.26 mg to 7.58 mg, as well as challenges with 40.5 mg of aspirin on the first day. On the second day of desensitization, patients were given aspirin in increasing doses from 81 mg to 325 mg. Patients were finally given 650 mg aspirin on the third day of the protocol. Blood samples were collected before the first desensitization challenge, at the time of the first clinician-confirmed reaction, and after the final desensitization challenge.

Clinical outcomes

Patient AERD symptom scores (score range 0-24) were calculated by using a scoring system as previously described at the time of reaction during aspirin challenge.[E1] Patient-reported 22-Item Sino-Nasal Outcome Test (SNOT-22) scores were also recorded at the time of aspirin challenge reaction (score range 0-110).

Nasal ILC2 identification

Nasal scrapings were performed with 1 to 3 passes over the inferior turbinate on 1 side with a Rhino-probe (Arlington Scientific, Springville, Utah), and each sample was placed in RPMI medium for processing for fluorescence-activated cell sorting analysis to detect ILC2s. After Fc blocking, cells were stained with peridinin-chlorophyll-protein–conjugated CD45 (Biolegend), fluorescein isothiocyanate–conjugated lineage markers (CD235a, FcεR1α, T-cell receptor γ/δ, and CD4 [Biolegend]), and BD Lineage Cocktail 1 (CD3, CD14, CD16, CD19, CD20, and CD56) (BD Biosciences, San Jose, Calif) and phycoerythrin-conjugated CRTH2 (Miltenyi Biotec, Bergisch Gladbach, Germany). ILC2s were defined as $CD45^+$ lineage–negative $CRTH2^+$ lymphocytes, as we have previously described.[E1,E2] Percentages of ILC2s are reported as percentages of total cells in the nasal scraping sample. Flow cytometry was performed using a Novocyte cytometer (Acea Biosciences, San Diego, Calif). Data were further analyzed with FlowJo software (FlowJo LLC, Ashland, Ore).

Lipidomic analysis

Patient serum samples were collected at baseline before desensitization, at the time of aspirin challenge reaction, and after desensitization, and a lipidomic panel for 161 eicosanoids was performed. A complete list of the eicosanoids included in this panel, including their identifiers and systematic names, is available on request. The 15-HETE presented in the article text and figures refers to 15-(S)-HETE. Eicosanoids were isolated from serum samples by using a Strata-X polymeric reverse phase column (Phenomenex, Torrance, Calif). Samples were run on an Acquity Ultra Performance Liquid Chromatography system and analyzed via mass spectrometer (Sciex 6500 Qtrap). Lipid analysis was performed at the University of California San Diego Lipidomics Core.

Statistics

Statistical analysis was performed by using RStudio (R, version 4.0.0) and GraphPad Prism (GraphPad Software, San Diego, Calif). Correlations between the lipid mediators and airway ILCs and clinical scores were calculated using RStudio. The following packages were utilized for this analysis: Hmisc, PerformanceAnalytics, ggcorrplot, tmvnsim, mnormt, and psych. Correlations were calculated by using the Spearman method and the package pairwise.complete.obs. GraphPad Prism software was used to plot correlations of interest. Goodness-of fit-and *P* values were calculated by using simple linear regression. *P* values less than 0.05 were considered statistically significant.

REFERENCES

E1. Eastman JJ, Cavagnero KJ, Deconde AS, Kim AS, Karta MR, Broide DH, et al. Group 2 innate lymphoid cells are recruited to the nasal mucosa in patients with aspirin-exacerbated respiratory disease. J Allergy Clin Immunol 2017;140:101-8.e3.

E2. Walford HH, Lund SJ, Baum RE, White AA, Bergeron CM, Husseman J, et al. Increased ILC2s in the eosinophilic nasal polyp endotype are associated with corticosteroid responsiveness. Clin Immunol 2014;155:126-35.
